# Maternal Plasma Procalcitonin Concentrations in Pregnancy Complicated by Preterm Premature Rupture of Membranes

**DOI:** 10.1155/2007/35782

**Published:** 2007-06-21

**Authors:** Andrzej Torbé

**Affiliations:** Department of Obstetrics and Gynecology, Pomeranian Medical University, Al. Powstańców Wielkopolskich 72, 70-111 Szczecin, Poland

## Abstract

*Objectives*. Our objective is to compare maternal plasma procalcitonin concentrations in preterm premature rupture of membranes (pPROM) and premature rupture of membranes (PROM) at term with their levels in uncomplicated pregnancy, and to determine whether these concentrations are useful in the diagnosis of pPROM cases suspected of infection and in the prediction of pPROM-to-delivery interval. Study design. Forty eight patients with pPROM, 30 with PROM at term, 31 healthy women at preterm gestation, and 33 healthy women at term were included. In pPROM group, analysis of procalcitonin concentrations with reference to leucocytosis, serum C-reactive protein, vaginal fluid culture, neonatal infection, histological chorioamnionitis and pPROM-to-delivery interval was carried out. *Results*. Procalcitonin concentrations in pPROM and PROM at term cases were comparable. However, in both groups procalcitonin values were significantly higher than in healthy controls in approximate gestational age. In pPROM group, procalcitonin concentrations between the patients with and without laboratory indices of infection were comparable, as well as between patients who gave birth to newborns with and without congenital infection, and between patients with and without histological chorioamnionitis. The predictive values of procalcitonin determinations were poor. *Conclusion*. The value of maternal plasma procalcitonin determinations in the diagnostics of pPROM cases suspected of intraamniotic infection, as well as for the prediction of pPROM-to-delivery interval, newborn's infection or histological chorioamnionitis is unsatisfactory. However, procalcitonin concentrations are elevated, both in patients with preterm and term PROMs in comparison to healthy pregnants, and therefore further evaluations are necessary to establish the role of procalcitonin in the pathophysiology of pregnancy.

## 1. INTRODUCTION

The management of a patient who presents with preterm premature rupture of membranes (pPROM) is controversial and remains a challenging task in perinatal medicine. In term patients, labor may be induced immediately after PROM or they may be observed for up to 24–72 hours for the onset of spontaneous labor [1, 2]. Clinical management of pPROM before 34 weeks of gestation is generally expectant, with controversies sorrounding the use of amniocentesis, corticosteroids, and tocolytics. In women without signs of infection, the current standard of care is hospitalization and bed rest until there is evidence of ascending infection or documentation of fetal lung maturity [3, 4].

Primary intraamniotic subclinical infection is one of the main causes of pPROM and the early identification of such cases is necessary for choosing the proper mode of management [3, 5]. However, the number of methods of detecting subclinical infection is modest and limited. Several studies have demonstrated that fetal compromise can be suspected by measuring inflammatory mediators not only in amniotic fluid or in cervicovaginal secretion, but also in the maternal blood.

Recently procalcitonin (PCT) was acknowledged as a specific marker of generalized bacterial infections [6, 7]. In serum of healthy individuals, it is present at less than 0.5 ng/mL; but in conditions leading to systemic inflammatory response syndrome or sepsis, it rapidly increases to high levels [8–10]. Although PCT continues to be found increasingly useful in modern clinical practice, there are only a few published data on PCT in pregnancy [11–13]. In our own recent study, higher plasma PCT concentrations were found in preterm labor when compared to healthy pregnants [12].

The purpose of the present study was

(1) to evaluate and compare plasma PCT concentrations in pregnancies complicated by pPROM and PROM at term with their levels in uncomplicated pregnancy,

(2) to determine whether maternal plasma PCT concentrations are of value in the diagnosis of pPROM cases suspected of subclinical intraamniotic infection (IAI) and in the prediction of the length of the pPROM-to-delivery interval.

## 2. MATERIAL AND METHODS

A total of 142 women with singleton pregnancies was enrolled in this study after providing written informed consent. The study was approved by the University Human Subject Review Committee. Gestational age was based on the last menstrual period and was confirmed by early second-trimester ultrasonographic examination. The study population was divided into four groups and the demographic characteristics of them are shown in Table 1.

Women with pPROM between 24 and 34 weeks of pregnancy without uterine contractions made up the first group. None of them showed clinical signs of infection or any other maternal or fetal complications at admission. All patients received antibiotics, steroids, and were restricted to bed rest with fetal heart monitoring and uterine activity assessment performed twice daily. The second group consisted of 30 women with PROM > 36 weeks without uterine contractions, and none of them had other diseases or obstetric complications. The induction of labor with intravenous oxytocin was initiated if spontaneous contractions did not ensue within 6–12 hours (*n* = 18; 60%). For PCT evaluations, in all patients with PROM, venous blood was collected within 4 hours, before administration of any drugs. In both groups, white blood cell count (WBC) and C-reactive protein (CRP) were analyzed, also within 4 hours of PROM. The third group consited of 31 healthy women at preterm gestation, and the fourth group consisted of 33 healthy women at term not in labor, with intact membranes. In these patients, venous blood was collected during routine ambulatory visits.

In all instances, maternal venous blood collected to the tubes containing EDTA was immediately centrifuged and then plasma was stored at −35°C until analysis. For the measurement of PCT, an immunoluminometric assay was performed using the LUMItest PCT kit (Brahms Diagnostica, Berlin, Germany) and the luminometer LIA-MAT System 300 (BYK-Sangtec Diagnostica, Dietzenbach, Germany). The lowest detection limit of PCT with this method was 0.1 ng/mL.

In the pPROM group, a detailed analysis of PCT concentrations was carried out and the results were compared to laboratory indices of subclinical IAI, the presence/absence of neonatal congenital infection, or the presence/absence of histological chorioamnionitis. The studied patients were also categorized into those who delivered within and after 24 and 72 hours after pPROM.

WBC was determinated with Celldyn 1700 and 3500 Abbott instruments (Abbott Laboratories, Ill, USA) with a critical value for analysis over 15.0 G/L. CRP was measured by immunoturbidometry with the Olympus AU 560 system (Olympus Diagnostica, Hamburg, Germany) with a critical value for analysis over 10 mg/L. In the pPROM group, vaginal fluid was cultured for aerobic and anaerobic bacteria. Fetal outcome was evaluated by a neonatologist according to clinical signs and laboratory tests of the neonate. Neonatal infection was assumed to be perinatally acquired if it occured within 48 hours after delivery. For the diagnosis of histologic chorioamnionitis, microscopic analysis of the placenta was performed.

Statistical analysis was performed with Statistica 5.5 and MedCalc 7.2 software. The Shapiro-Wilk test was used to check the distribution of analyzed parameters. Differences between the groups were analyzed by Mann-Whitney *U*. test and Chi^2^ test. Possible correlation between plasma PCT at the onset of pPROM and WBC count, CRP level, gestational age, and the length of pPROM-to-delivery interval were assessed by Spearman's test. Receiver operator characteristic curve analysis was used to establish the cutoff value of plasma PCT that optimized the prediction of pPROM-to-delivery interval, neonatal infection, and histological chorioamnionitis. Sensitivity, specificity, positive and negative predictive values were then calculated. Additionally, the predictive values of WBC count ≥15.0 G/L and plasma CRP ≥10 mg/L were evaluated.

## 3. RESULTS

Plasma PCT concentrations in patients with pPROM and with PROM at term were comparable (Table 2). Also WBC count (12.45 versus 11.35 G/L; *P* = .84) and CRP levels (8.0 versus 6.6 mg/L; *P* = .65) were comparable. However, in both groups, PCT values were significantly higher in comparison to healthy controls in approximate gestational age (Table 2).

The pregnancies complicated by pPROM were subjected to particulary careful analyses. Laboratory indices suggesting the presence of subclinical IAI were searched by evaluating WBC count, CRP concentration, and vaginal fluid culture, however in these cases no clinical signs of infection were found (Table 3). In 9 (18.75%) positive cultures of vaginal fluid, *Candida albicans* was found in 7 cases and *Streptococcus* B was found in 2 cases.

Although in 29 (60.42%) cases of pPROM, at least one laboratory parameter suggesting the presence of subclinical IAI was observed, the differences in PCT concentrations between the patients with and without laboratory indices of infection were not significant (Table 3). Also, no correlation was observed between PCT concentration and WBC count (*r* = . 01; *P* = .93) or CRP level (*r* = −.14; *P* = .36).

Out of 48 patients enrolled in the pPROM group, 17 (35.42%) neonates developed infection, but plasma PCT concentrations of their mothers (Table 3), and their mothers' WBC counts (13.60 versus 12.10 G/L; *P* = .10) were comparable to those in women whose newborns were healthy. Only plasma CRP concentrations of mothers who gave birth to newborns with congenital infection were significantly higher (12.80 versus 7.40 mg/L; *P* = .01).

In 14 (29.17%) placentas, inflammatory changes were found, however in these patients, PCT concentrations (Table 3), as well as WBC count (14.40 versus 12.10 G/L; *P* = .47) and CRP levels (8.67 versus 4.91 mg/L; *P* = .41) were also compatible to those without changes.

The cutoff value for PCT against newborn's infection or histological chorioamnionitis showed that the highest sensitivity and specificity corresponded to a concentration of 1.9 ng/mL, but the predictive values were generally poor, as well as the predictive values of WBC count and CRP levels (Table 4).

No correlations were observed between PCT concentrations at the moment of pPROM and gestational age at this time (*r* = .07; *P* = .66). In cases in which regular uterine contractions appeared spontaneously (*n* = 33), the relationship between maternal PCT concentrations and the length of pPROM-to-delivery interval was analyzed, but it failed to reveal significant correlation (*r* = .14; *P* = .35).

Comparison of PCT concentrations in cases delivered, after the spontaneous appearance of contractions, within 24 hours and 3 days of pPROM and after these times also did not show significant differences (Table 3). The highest values in the prediction of delivery within 24 and 72 hours corresponded to a PCT concentration of 1.9 ng/mL, but they were unsatisfactory (Table 4).

## 4. COMMENT

The early identification of IAI is a desirable goal in patients with term, or particularly with preterm amniorrhexis. Clinical signs of infection are subtle and usually not present in early chorioamnionitis. Currently, there are no reliable clinical markers to adequately indicate IAI in these patients. Numerous studies have demonstrated that it can be suspected by measuring various mediators in amniotic fluid. Although these diagnostic methods are highly accurate, they require the performance of amniocentesis, which regrettably is an invasive technique, and may be difficult to perform when the amniotic fluid volume is significantly reduced. Therefore, alternative methods to assess the microbial status of the intrauterine environment, indirectly but noninvasively, are proposed. The maternal serum is an easy approach compartment which offers the possibility of obtaining biological material in almost noninvasive way. The usefulness of various markers in the management of pPROM cases, such as proinflammatory cytokines, CRP, WBC, neutrophil counts, granulocyte elastase, ferritin or glycodelin, was previously evaluated [4, 14–20]. Recently, Assi et al. [21] described a significant increase of maternal plasma concentrations of pentraxin 3, which is an inflammatory molecule belonging to the family of CRP, in pregnancies complicated by preterm delivery.

PCT is a new parameter used in the diagnosis of generalized or systemic infectious diseases. In view of the probably infectious etiology of PROM, one of the purposes of this study was to determine whether maternal plasma PCT concentrations might have any value in the diagnosis and management of these cases.

The first goal of this study was to determine whether maternal serum PCT concentrations between pPROM and PROM at term and healthy pregnants are different or comparable. It was observed that PCT levels in pPROM were comparable to those in cases of amniorrhexis at term. However, in both situations they were signifintly higher than in cases without complications of the same gestational week, which may represent additional evidence confirming the hypothesis about the infectious etiology of rupture of the membranes.

The pregnancies complicated by pPROM were subjected to particulary careful analyses. However, we did not observe clinical signs of infection in any of the cases, in over 60% at the onset of pPROM, at least one laboratory parameter suggesting the presence of subclinical IAI was observed and over 35% neonates developed congenital infection. Our results showed that plasma PCT concentrations do not correlate significantly with laboratory indices suggestive of sublinical infection and that PCT values do not differ between subgroups of women with and without laboratory indices of infection at the onset of pPROM. A cutoff value of ≥1.9 ng/mL predicted neonatal infection, histological chorioamnionitis, or delivery within 24 and 72 hours from pPROM with a sensitivity of 53%, 75%, 55%, 48%, and with a specificity of 45%, 45%, 55%, 50%, respectively.

In conclusion, our findings suggest that the value of maternal plasma procalcitonin determinations in the diagnostics of pPROM cases suspected of subclinical IAI, as well as for the prediction of pPROM-to-delivery interval, newborns infection or histological chorioamnionitis is unsatisfactory. However, PCT concentrations are elevated, both in patients with preterm and term PROMs in comparison to healthy pregnants, and therefore further evaluations are necessary to establish the role of PCT in the pathophysiology of pregnancy.

## Figures and Tables

**Table 1:** Clinical characteristics of the groups.

Clinical charecteristics	Group 1	Group 2	Group 3	Group 4
	(*N* = 48)	(*N* = 30)	(*N* = 31)	(*N* = 33)

Maternal age (years) (mean ± SD)	27.5 ± 6.8	27.8 ± 5.3	26.7 ± 4.9	28.2 ± 5.2
Primiparous (*N*%)	22 (45.8)	15 (50.0)	16 (51.6)	16 (48.5)
Preterm delivery in prior pregnancy (*N*%)	11 (22.9)	2 (6.2)	4 (12.9)	1 (3.0)
Gestational age at PROM (weeks) (mean ± SD)	30.8 ± 3.3	38.6 ± 1.3	—	—
Gestational age at delivery (weeks) (mean ± SD)	31.4 ± 3.0	38.6 ± 1.3	38.9 ± 1.4	40.4 ± 1.0
pPROM-to-delivery interval (days) (mean ± SD)	5.5 ± 8.1	0	—	—
Cervical dilatation (cm) (mean ± SD)	1.9 ± 1.0	0.87 ± 0.8	0.4 ± 0.6	1.8 ± 0.8
Cervical length (cm) (mean ± SD)	1.4 ± 0.7	2.5 ± 1.1	1.8 ± 0.3	1.4 ± 0.5
Caesarean section delivery (*N*%)	26 (54.2)	5 (16.7)	7 (22.6)	10 (30.3)
Birth weight (g) (mean ± SD)	1828 ± 657	3412 ± 398	3515 ± 467	3650 ± 603
Five-minute Apgar score (points) (mean ± SD)	8.5 ± 1.4	9.7 ± 0.6	9.7 ± 0.6	9.5 ± 0.8

**Table 2:** Comparison of maternal plasma procalcitonin (PCT) concentrations between the groups.

Groups	*n*	PCT (ng/mL): median

Group 1: pPROM	48	1.97* ^†^
Group 2: PROM	30	1.60* ^‡^
Group 3: healthy-preterm gestation	31	1.06^†^ ^Δ^
Group 4: healthy at term	33	0.71^‡^ ^Δ^

Comparison between groups:

*1 and 2: NS

^†^1 and 3: *P* = .002

^‡^2 and 4: *P* = .045

^Δ^3 and 4: NS

**Table 3:** Plasma procalcitonin (PCT) concentrations of pregnant women with pPROM in relation to the presence of indicators of the infection, newborn status, histological chorioamnionitis, and pPROM-to-delivery time.

		*N*	PCT (ng/mL): median	*P* value

Leucocytosis	≤15.0 G/L	35	2.11	.40
	>15.0 G/L	13	1.51	

CRP	≤10 mg/L	30	1.61	.50
	>10 mg/L	18	2.21	

Vaginal fluid culture	Negative	41	1.71	.19
	Positive	7	3.15	

Newborn	Healthy	31	2.01	.35
	Infected	17	1.92	

Placenta	Lack of changes	34	2.13	.67
	Inflammatory changes	14	2.83	

Delivery	Within 24 hours	11	2.14	.84
	After 24 hours	22	1.49	

Delivery	Within 72 hours	21	1.71	.57
	After 72 hours	12	2.31	

**Table 4:** The prognostic value of maternal plasma procalcitonin (PCT), white blood cell (WBC) count, and C-reactive protein (CRP) determinations in the prediction of newborn's congenital infection, histological chorioamnionitis, and pPROM-to-delivery interval. PPV denotes positive predictive value; NPV denotes negative predictive value.

		Congenital	Histological	Delivery	Delivery
		infection	chorioamnionitis	within 24 hours	within 72 hours

PCT ≥1.9 ng/mL	Sensitivity (%)	53	75	55	48
	Specificity (%)	45	45	55	50
	PPV (%)	35	35	38	63
	NPV (%)	64	82	71	35

WBC ≥15.0 G/L	Sensitivity (%)	48	33	29	28
	Specificity (%)	85	80	83	88
	PPV (%)	63	44	50	80
	NPV (%)	76	71	67	42

CRP ≥10 mg/L	Sensitivity (%)	52	33	35	34
	Specificity (%)	76	64	79	88
	PPV (%)	52	31	50	83
	NPV (%)	76	67	68	44
